# Proline Dehydrogenase Gene *TaProDH1* Positively Regulates Wheat Resistance to Stripe Rust Disease

**DOI:** 10.3390/jof12090655

**Published:** 2026-09-01

**Authors:** Haibin Zhao, Yue Li, Cao Zhong, Nana Li, Qiang Xu

**Affiliations:** 1 Engineering Research Center of Microbial Resources Development and Green Recycling of Shaanxi Province, Shaanxi Key Laboratory of Research and Utilization of Resource Plants on the Loess Plateau, Yan’an University, Yan’an 716000, China; zhaohaibinxn1934@163.com (H.Z.); linana@yau.edu.cn (N.L.); 2 Triticeae Research Institute, Sichuan Agricultural University, Chengdu 625014, China; liyue1830338@163.com (Y.L.); zhongcaoyx@126.com (C.Z.)

**Keywords:** *TaProDH1* gene, *Triticum aestivum*, HIGS, TaMYB30, resistance

## Abstract

Proline dehydrogenase (ProDH) is a rate-limiting enzyme in the proline metabolism cycle that plays an important role in plant stress response and disease resistance. However, the function and mechanism of *ProDH1* in the wheat–*Puccinia striiformis* f. sp*. tritici* (*Pst*) interaction remain poorly understood. In this study, a wheat proline dehydrogenase gene *TaProDH1* was induced by stripe rust. qRT-PCR analyses showed that the transcript levels of *TaProDH1* were highly increased during early infection. The transient expression of *TaProDH1* in *Nicotiana benthamiana* leaves revealed that TaProDH1 increases the content of proline in leaves and induces the accumulation of reactive oxygen species. Silencing *TaProDH1* via the barley stripe mosaic virus (BSMV)-induced gene silencing (VIGS) system led to compromised wheat resistance to the *Pst* avirulent pathotype CYR23, significantly increased proline content in the plant, and increased mycelial growth and sporulation of the pathogen. In addition, it was confirmed by the yeast one-hybrid (Y_1_H) and dual-luciferase reporter systems that the transcription factor TaMYB30 can activate its transcriptional activity by binding to the *TaProDH1* promoter region. In summary, the *TaProDH1* gene is a positive regulator of stripe rust resistance in wheat. It is activated by the upstream transcription factor TaMYB30 and accelerates the process of proline metabolism, which is conducive to enhancing the resistance of wheat to stripe rust.

## 1. Introduction

*Triticum aestivum*, as a major food crop, is constantly under severe threat from pests and diseases, which has a significant impact on global food security. The filamentous fungus *Puccinia striiformis* f. sp. *tritici* (*Pst*) is the causative agent of wheat stripe rust, one of the world’s most destructive crop diseases that is estimated to result in annual losses of 1 billion US dollars [1]. Selecting resistant varieties is the most economical and effective measure for controlling diseases and achieving sustainable green development in agriculture [2,3]. However, the frequent mutations of the stripe rust fungus, leading to the loss of plant resistance, are the main reason for the prevalence and outbreak of this disease. Therefore, it is particularly important to conduct in-depth research on the mechanism of wheat’s resistance to stripe rust.

Plants have evolved a two-layered immune system to defend against pathogens, consisting of pattern-triggered immunity (PTI) and effector-triggered immunity (ETI) [4]. PTI is initiated by the perception of conserved pathogen-associated molecular patterns, such as flagellin and chitin, via plasma membrane-localized receptor complexes exemplified by the flg22 receptor [5]. This recognition subsequently activates mitogen-activated protein kinase (MAPK) signaling cascades, leading to callose deposition and rapid reactive oxygen species (ROS) bursts at infection sites. By contrast, ETI is mediated by intracellular nucleotide-binding site and leucine-rich repeat (NBS-LRR) immune receptors that recognize secreted pathogen effectors, ultimately triggering hypersensitive cell death to restrict pathogen colonization [6]. Plant immunity functions as a holistic system, wherein PTI and ETI are not mutually independent but contribute to one another [7,8].

Proline metabolism serves as a core regulatory pathway underlying plant stress adaptation, and the homeostasis of proline synthesis and degradation pathways exerts a decisive effect on plant resistance to biotic and abiotic stresses [9,10]. One major proline biosynthetic route is the glutamate pathway. In this pathway, glutamate is converted into glutamic acid-γ-semialdehyde (GSA) catalyzed by Δ^1^-pyrroline-5-carboxylate synthetase (P5CS), which is subsequently reduced to proline by pyrroline-5-carboxylate reductase (P5CR) [11]. This pathway dominates proline biosynthesis in plants and is markedly induced by abiotic stresses such as drought and salinity [10]. The second proline biosynthetic pathway is the ornithine pathway. Ornithine is converted into glutamic acid-γ-semialdehyde under the catalysis of ornithine aminotransferase (OAT), which is further converted to proline. This pathway predominates in tissues with active nitrogen metabolism, such as root tips [12]. The catabolic pathway involves proline dehydrogenase, which catalyzes the oxidation of proline to GSA, accompanied by the production of NADH and ROS. Subsequently, GSA is converted into glutamate under the action of Δ^1^-pyrroline-5-carboxylate dehydrogenase (P5CDH) [13].

Proline dehydrogenase (ProDH), a key enzyme governing proline metabolism in plants, functions critically in stress responses. It catalyzes the oxidation of proline into pyrroline-5-carboxylate (P5C) to facilitate proline degradation, thereby executing regulatory effects. ProDH-mediated proline metabolism engages in immune regulation through two distinct molecular mechanisms. One mechanism operates through the P5C–ROS signaling cascade. P5C derived from proline degradation undergoes further oxidation to glutamate in the mitochondria, accompanied by the generation of NADH and ROS. The resulting transient ROS burst activates MAPK signaling cascades (e.g., MPK3/6) as well as the NPR-dependent SA pathway, thereby inducing the expression of defense genes such as *PR1* [14]. The second mechanism involves α-ketoglutarate-mediated epigenetic regulation. Glutamate generates α-ketoglutarate via transamination. As an essential cofactor for TET family dioxygenases, α-ketoglutarate may remodel the chromatin state of disease resistance genes by modulating DNA demethylation or histone modifications [15]. Indirect evidence supporting this regulatory model comes from the *Arabidopsis thaliana*–*Botrytis cinerea* interaction system: *AtProDH1* mutants display substantially reduced levels of H_3_K_4_me3 modifications at the promoter regions of defense genes [16]. Recent studies have revealed that ProDH modulates cellular redox status by maintaining proline metabolic homeostasis; furthermore, it directly participates in the establishment of plant immune networks via its metabolites and ROS signaling. In *Arabidopsis thaliana*, the *AtProDH1* and *AtProDH2* genes encode functionally non-redundant proline dehydrogenase isoforms with distinct biological functions [17]. These observations indicate that ProDH activates systemic acquired resistance (SAR) via mitochondrial ROS production [18]. By contrast, in the *Arabidopsis*–*Sclerotinia sclerotiorum* pathosystem, *AtProDH2*-overexpressing plants exhibit an unrestrained ROS burst triggered by excessive proline degradation, which accelerates programmed cell death (PCD) [19].

In this study, we report the role of a wheat proline dehydrogenase, TaProDH1, in the interaction between wheat and stripe rust fungus. We found that TaProDH1 was significantly induced in wheat leaves when challenged by *Pst* fungus. Agrobacterium-mediated transient expression of *TaProDH1* increased the content of proline and induced ROS accumulation in *N. benthamiana*. Furthermore, the host-induced gene silencing (HIGS) of *TaProDH1* compromised wheat resistance to the *Pst* avirulent, thereby increasing the proline content of the plant and the mycelial growth and sporulation of the pathogen. In addition, transcription factor TaMYB30 was found to activate its transcriptional activity by binding to the TaProDH1 promoter region. These results shed light on the contributions of TaProDH1 to wheat resistance against stripe rust. TaProDH1 may function as a positive regulator in defense responses by increasing the content of proline and the accumulation of ROS during the early stages of *Pst* infection.

## 2. Materials and Methods

### 2.1. Plant Materials, Fungal Isolates and Inoculation Treatments

Tobacco (*N. benthamiana*), wheat cultivar Suwon11 (Su11), MX169 and *Pst* isolates CYR23 (avirulent), and CYR34 (virulent) were used in this study. Wheat cultivar MX169 was used to propagate *Pst* races CYR23 and CYR34, following the methods described in previous reports [20]. Wheat seedlings were cultured at 16 °C with a standard 16-h light/8-h dark cycle. *N. benthamiana* was cultured at 25 °C with a photoperiod of 16 h of light and 8 h of darkness. The *Saccharomyces cerevisiae* strain Y187 was used in this study.

### 2.2. Identification, Cloning and Sequence Analysis of TaProDH1

A 1443 bp nucleotide sequence (accession number: TraesCS1D02G212400) was obtained from the cDNA library in our lab. The coding sequence (CDS) of *TaProDH1* was amplified from the cDNA template obtained from Su11. The PCR program was as follows: 3 min at 95 °C, followed by 35 cycles of 30 s at 95 °C, 30 s at 60 °C, and 60 s at 72 °C, and finally 10 min at 72 °C. The PCR mixture contained the following: 20 μL of 2×Es taq MasterMix, 1 μL of template, and 1 μL of each primer. Next, the total volume was made up to 15 μL with ddH_2_O. The target fragments were inserted into the pGEM-T Easy vector (Promega, Madison, WI, USA) and sequenced. The TaProDH1 sequence was used to run a BLAST+v2.13.0 search on the WheatOmics website and the Ensembl plant database at http://plants.ensembl.org/index.html (accessed on 16 July 2026). The conserved domain was predicted with Pfam (http://pfam.sanger.ac.uk/) and InterproScan (http://www.ebi.ac.uk/Tools/pfa/iprscan/). DNAMAN8.0 was used to perform sequence alignments.

### 2.3. RNA and Real-Time PCR (qRT-PCR) Analysis

Wheat seedlings at the two-leaf stage were inoculated with CYR23 and CYR34. The wheat leaves were sampled at 0, 24, 48, 72, and 120 h post inoculation (hpi) and immediately frozen in liquid nitrogen and stored at −80 °C before extraction of total RNA. Then, total RNA was extracted with the TransZol Up Plus RNA Kit was purchased from TransGen Biotech Co., Ltd., Beijing, China and the operation method used was taken from the product manual instructions. Next, cDNAs were synthesized using the RevertAid First Strand cDNA Synthesis Kit was purchased from Thermo Fisher Scientific, Waltham, MA, USA.

The *TaProDH1*, *TaPR1*, *TaPR2*, and *TaPR4* transcript levels were analyzed using qRT-PCR. *TaEF* and *PstEF* were used as internal controls for wheat and *Pst*, respectively. qRT-PCR was conducted on a CFX Connect Real-time instrument (Bio-Rad, Hercules, CA, USA), following the manufacturer’s instructions. Three biological replicates were prepared for each sample. All qPCR amplifications were performed in 20 μL reaction mixtures containing 10 μL of ChamQ Universal SYBR qPCR Master Mix (Vazyme Biotech Co., Ltd., Nanjing, China, Q711-02), 10 pmol of primers, and 2 μL of diluted cDNA (1:10). Relative expression levels of target genes were calculated using the comparative 2^−ΔΔCT^ method.

### 2.4. Transient Overexpression of TaProDH1 in N. benthamiana

The CDS of TaProDH1 was constructed into the GFP-tagged pCAMBIA1302 vector. The empty pCAMBIA1302 vector and recombinant TaProDH1-GFP plasmid were transformed into *Agrobacterium tumefaciens* strain GV3101, which was incubated at 28 °C for 3 days. The bacteria were harvested by centrifugation and resuspended in the acetosyringone (AS) buffer containing 10 mM MES (pH 5.6), 10 mM MgCl_2_, and 150 μm acetosyringone, after being washed with 10 mM MgCl_2_. Strains carrying different recombinant vectors were adjusted to a density of 0.6 at 600 nm (OD_600_) with AS buffer and incubated for 2 h in the dark at room temperature before leaf infiltration. These leaves were sampled at 1, 2, and 3 days post-infiltration (dpi) and stained with 3,3′-diaminobenzidine (DAB) or trypan blue [21]. ROS content was determined based on the luminol/horseradish peroxidase (HRP) detection principle [22]. The pCAMBIA1302-GFP construct was used as a control. These experiments were repeated at least three times and yielded the same result.

### 2.5. Measurement of Proline Content

To determine the proline content in *N. benthamiana* and wheat under different treatments, we plotted a standard curve and further measured the proline levels in samples [23].

### 2.6. BSMV-Mediated Gene Silencing of TaProDH1

Silencing of *TaProDH1* was achieved using BSMV-mediated gene silencing method, as previously described [24]. BSMV:γ was used as the negative control, and BSMV:TaPDS was used as a virus-positive control. Ten days after virus inoculation, the fourth leaves were further inoculated with the fresh urediniospores of *Pst* CYR23. The disease phenotypes in BSMV-challenged wheat leaves were monitored and recorded at 14 dpi. *Pst*-wheat leaves were sampled at the indicated time points for further analysis. The leaves were sampled at 0, 24, 48, and 120 hpi for histological changes and analyzed by qRT-PCR.

### 2.7. Subcellular Localization of TaProDH1 and TaMYB30

To clarify the subcellular localization of TaProDH1, the CDS of *TaProDH1* was cloned into the pCAMBIA1302 and pTF486 vectors to generate the recombinant pCAMBIA1302-*TaProDH1* plasmid and pTF486-*TaProDH1*. Subsequently, pCAMBIA1302-*TaProDH1* and TaAIF-RFP carrying a mitochondrial localization signal were individually transformed into the *A. tumefaciens* strain GV3101 and injected into 5-week-old *N. benthamiana* leaves [25]. Furthermore, the recombinant pTF486-*TaProDH1* plasmid was transformed into wheat protoplasts, according to previously described methods [26]. pCAMBIA1302 and pTF486 were used as empty vector controls. A similar experimental protocol was used for the subcellular localization analysis of TaMYB30.

### 2.8. Yeast One-Hybrid Assay (Y1H)

Construction of the pHis2-*TaProDH*1-C1-Pro (983–2000 bp) vector included vector construction in *Escherichia coli* and subsequent plasmid extraction. The recombinant plasmid pHis2-*TaProDH1*-C1-Pro was transformed into competent Y187 yeast cells, followed by incubation in an incubator at 28 °C for 2–3 days to identify the transformation outcome.

Self-activation assay of the promoter of *TaProDH1*: Prepare SD-Trp/His medium containing a series of 3AT concentrations ranging from 0 to 100 mM. Pick single yeast colonies of pHis2-*TaProDH1-C1*-Pro grown on SD-Trp plates, inoculate them into SD-Trp liquid medium, and incubate overnight at 28 °C with shaking until the OD value reaches 0.2. Dilute the bacterial suspension with sterile water to OD values of 0.02, 0.002 and 0.0002 sequentially, then spot the diluted suspensions onto SD-Trp/His plates with different 3-AT concentrations. The optimal working concentration of 3-AT is determined based on the screening result of the yeast suspension at an OD value of 0.0002.

Screening assay: Competent cells were prepared from Y187 yeast cells harboring the pHis2-*TaProDH1-C1*-Pro plasmid, followed by co-transformation with library plasmids. Two plates of SD-Trp/His medium supplemented with 3.5 mM 3-AT (the optimal concentration to suppress autoactivation of the 983–2000 bp promoter fragment) and thirty plates of SD-Trp/His/Leu medium containing 3.5 mM 3-AT (the optimal concentration to suppress autoactivation of the 983–2000 bp promoter fragment) were prepared. The plates were incubated at 28 °C for 3–5 days. Larger putative positive yeast colonies were subjected to positive verification, and subsequent sequencing was performed to obtain candidate interacting genes.

### 2.9. Verification of Transcriptional Activation by TaMYB30 Protein

The three recombinant plasmids, pGBKT7-*TaMYB30*, pGBKT7*-TaMYB30*_1–120_, and pGBKT7-*TaMYB30*_121–254_, were directly transformed into the DH5α *E. coli* strain and amplified. Subsequently, the pGBKT7-*TaMYB30* recombinant plasmids, the pGBKT7-*Lam* plasmid (negative control), and the pGBKT7-*p53* (positive control) were introduced into the AH109 yeast strain containing the *HIS3*, *ADE2*, *MEL1*, and *lacZ* reporter genes. The transformed yeast strains were plated on a medium without tryptophan (SD/-Trp) and incubated at 30 °C for 2 to 3 days. The positive clones on the Trp-deficient media were selected and transferred into yeast peptone dextrose adenine (YPDA) liquid media for culturing. Next, the yeast solutions were diluted to OD_600_ of 1, 10^−1^, 10^−2^, and 10^−3^. Lastly, these solutions were inoculated into a medium lacking adenine, histidine, and tryptophan (SD-His/-Ade/-Trp) and supplemented with X-α-D-galactoside (X-α-Gal). These cultures were incubated at 30 °C for 3 days.

### 2.10. Yeast Two-Hybrid Assay (Y2H)

To analyze the interaction of TaProDH1-C1-pro and TaMYB30 in yeast, the full-length coding sequences of *TaMYB30* and *TaProDH1-C1-pro* were inserted into the pGADT7 and pGBKT7 vectors, respectively. These recombinant vectors were transformed into yeast strain AH109 using the lithium acetate method, in accordance with the manufacturer’s instructions (Clontech Laboratories, Inc., Mountain View, CA, USA). Subsequently, transformants were grown on synthetic dropout agar medium (SD-Leu-Trp) and screened on SD/-Leu/-Trp/-His/-Ade medium supplemented with 3.75 mM 3-AT. Images were captured after 5 days of incubation at 30 °C.

### 2.11. The D-LUC Assay

The CDS of *TaMYB30* was inserted into the pGreenII-62SK vector, and the 2000 bp promoter sequence upstream of the start codon of the *TaProDH1* gene was cloned into the pGreenII-0800 vector. For the control group, tobacco leaves were co-infiltrated with Agrobacterium harboring the empty pGreenII-62SK vector and Agrobacterium carrying the TaProDH1-pGreenII-0800 construct. For the experimental group, tobacco leaves were co-infiltrated with Agrobacterium containing TaMYB30-pGreenII-62SK and Agrobacterium harboring *TaProDH1*-pGreenII-0800. Three groups of tobacco leaves of equal quantity and consistent growth status were sampled from each of the above two groups. The enzymatic activities of fLUC and rLUC were determined separately following the procedures described below, in accordance with the instructions supplied by the dual-luciferase reporter assay kit.

### 2.12. Histology of Fungal Infection and Host Response

To visualize fungal hyphae, wheat leaf tissues inoculated with *Pst* for 24 or 48 h were destained in 1 M KOH at 37 °C for 24 h. Samples were then washed three times with 50 mM Tris-HCl (pH 7.4) and stained for 24 h in the dark with wheat germ agglutinin. Hyphal length was quantified using CellSens Entry software v3.2 and an Olympus BX-51 microscope (Olympus Corporation, Tokyo, Japan). For each wheat leaf sample in each biological replicate, 30 infection sites were analyzed from three leaves collected from different plants. Infection sites were selected using a randomized block design with three replicates.

### 2.13. Statistical Analysis and Graphical Presentation

All data analyses were performed using GraphPad 8.0, and the statistical analyses were performed using SPSS 26.0. Significant differences between experimental and control groups were determined by two-tailed Student’s *t* tests and one-way ANOVA.

## 3. Results

### 3.1. Cloning and Sequence Analysis of Wheat ProDH1

Research on other plants suggests that the DH gene may be involved in plants’ responses to stress conditions. We analyzed the amino acid sequences of the three homologous genes of the *TaProDH1* gene in wheat (Figure S1). The target gene of this study, TraesCS1D02G212400, has the highest similarity to the TraesCS1B02G223300 gene sequence of the B genome. In the highly conserved proline dehydrogenase domain (121–459 aa), only the 237th amino acid contains a variation in arginine (R)-histidine (H). The amino acid sequence of TraesCS1A02G209100 has a deletion of 112 amino acids at the N-terminal. Compared with the proline dehydrogenase domain of TraesCS1B02G223300 and TraesCS1D02G212400, there are 12 amino acid differences. Therefore, due to the conservation of the amino acid sequence, we speculate that TraesCS1D02G212400 and TraesCS1B02G223300 may play significant roles in gene function.

### 3.2. TaProDH1 Is Localized in Mitochondria

**Figure 1:**
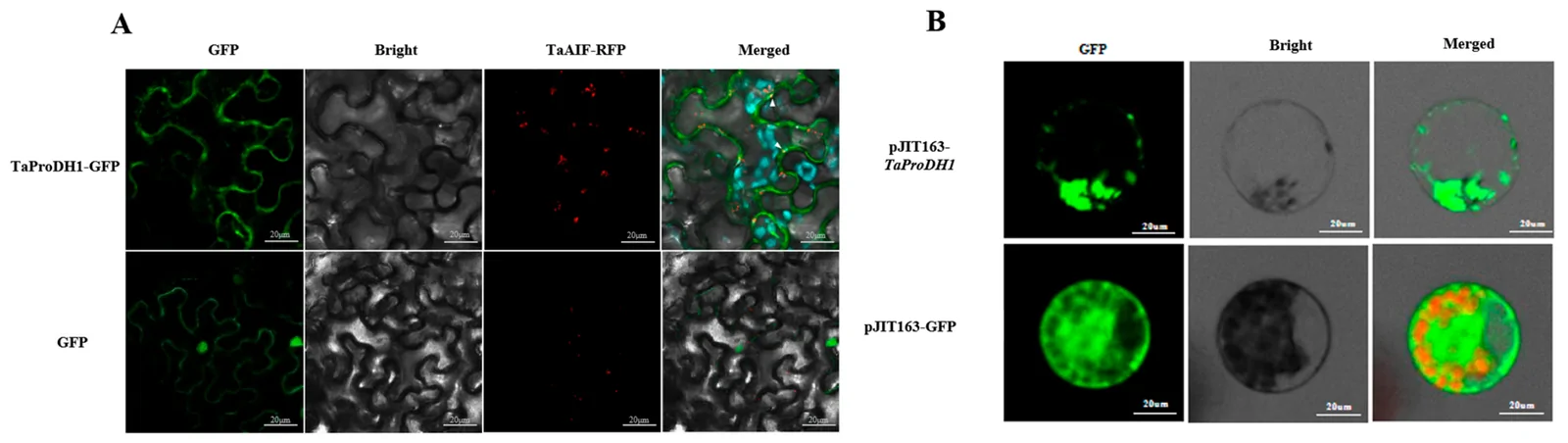
Subcellular localization of TaProDH1 in the plant. (**A**) Transient expression of free GFP or TaProDH1-GFP fusion protein in *N. benthamiana* leaves. The AIF (apoptosis-inducing factor) is known to localize to the mitochondria. (**B**) Subcellular localization of TaProDH1 in wheat protoplasts. pJIT163 vector carrying a green fluorescent protein (GFP) tag was used.

TaProDHs are mitochondria-targeting proteins [13]. To illustrate the subcellular localization of TaProDH1, the TaProDH1-GFP fusion protein and free GFP were transiently expressed in *N. benthamiana* leaves and wheat protoplasts (Figure 1), respectively. Furthermore, TaProDH1-GFP and TaAIF-RFP (the apoptosis-inducing factor (AIF) with known mitochondrial localization) were co-infiltrated into *N. benthamiana* leaves. The fluorescence signal was observed via laser scanning confocal microscopy. As expected, fluorescence signals from the free GFP were detected throughout the cytoplasm and nucleus, while the green fluorescence of TaProDH1-GFP partially colocalized with the red fluorescence of TaAIF-RFP (Figure 1A). Therefore, we conclude that TaProDH1 is a mitochondrial protein.

### 3.3. TaProDH1 Is Induced During Pst Infection in Wheat

**Figure 2:**
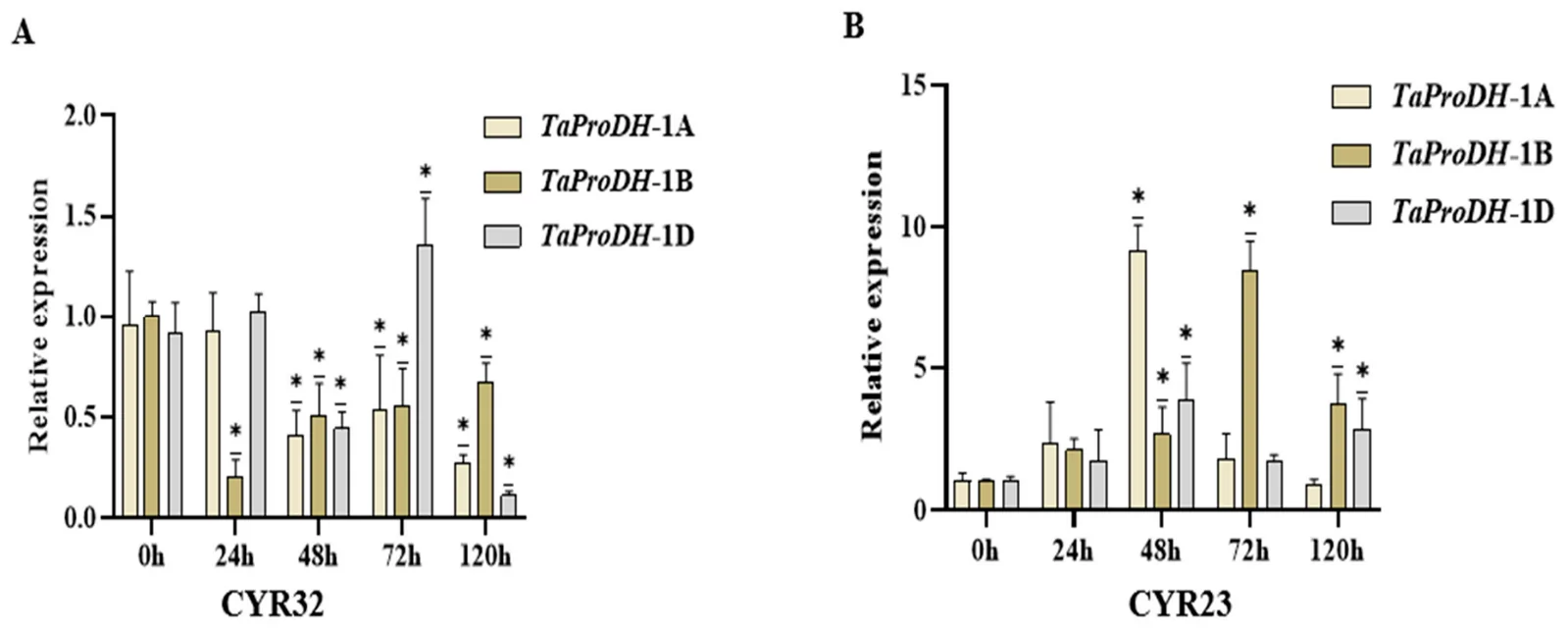
Analysis of expression characteristics of the TaProDH1 gene induced by stripe rust. (**A**) Relative expression levels of TaProDH1 in wheat leaves upon infection with CYR34; (**B**) relative expression levels of TaProDH1 in wheat leaves inoculated with CYR23. Seedling leaves of wheat cultivar SU11 at the two-leaf stage were inoculated with *Puccinia striiformis* f. sp. *tritici* races CYR23 and CYR34, respectively. Samples were collected at 0 h, 24 h, 48 h, 72 h, and 120 h post inoculation, with the 0 h time point set as the control. Gene expression levels were determined via qRT-PCR. 1A and 1B represent homologous genes of TaProDH1-1D. Error bars indicate standard deviations from three biological replicates, and asterisks indicate significant differences (*p* < 0.05).

We employed the expVIP platform (http://www.wheat-expression.com/, accessed on 16 July 2026) to investigate the gene expression profiles induced during the wheat–*Pst* interaction and revealed that TaProDH1 was significantly upregulated after inoculation with *Pst* (Figure S2). RT-qPCR was used to analyze the induced expression patterns of *TaProDH1* in wheat cultivar SU11 after inoculation with *Pst* races CYR23 and CYR34, respectively. The results showed that in the compatible interaction (Figure 2A), the expression of *TaProDH1-1D* was significantly induced upon inoculation. Its expression was markedly downregulated at 48 h and 120 h, while significantly upregulated at 72 hpi (Figure 2A). In the incompatible interaction (Figure 2B), *TaProDH1-1D* was obviously upregulated at 48 h and 120 h after inoculation. Its homoeologous gene *TaProDH1-1A* exhibited significant upregulation at 48 h post inoculation, and *TaProDH1-1B* was significantly upregulated at 48 h, 72 h and 120 h after inoculation. Collectively, *TaProDH1* may be involved in the resistance response of wheat to stripe rust.

### 3.4. TaProDH-Induced ROS Accumulation in N. benthamiana Leaves

**Figure 3:**
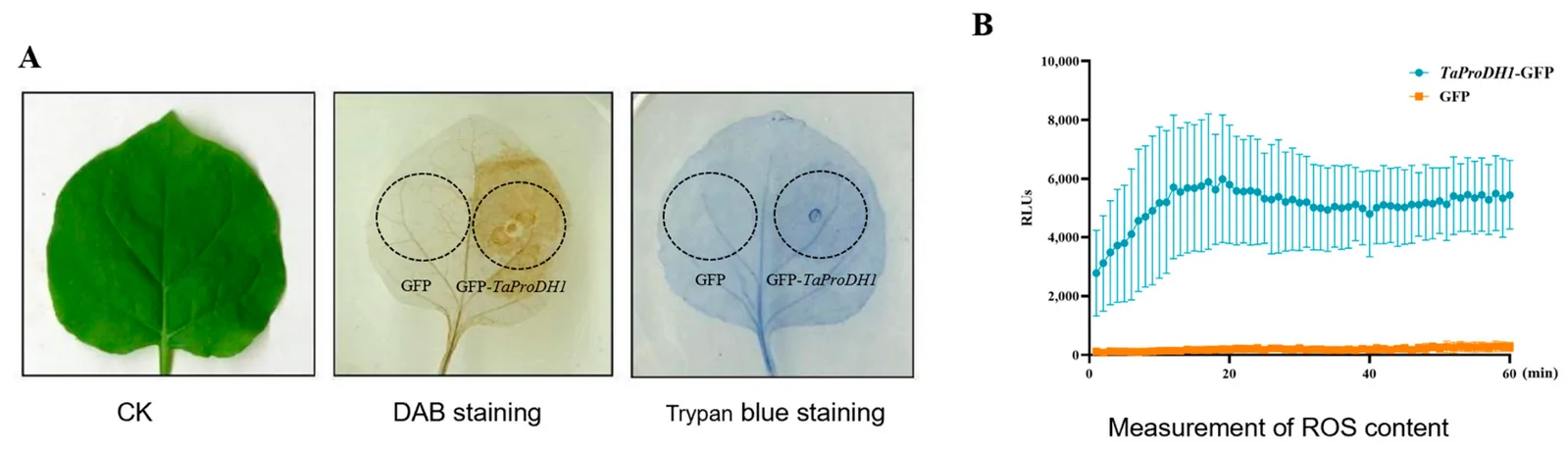
TaProDH1 stimulates reactive oxygen species in *N. benthamiana*. (**A**) DAB staining and trypan blue staining phenotypes of transiently expressed GFP and TaProDH1-GFP in *N. benthamiana* leaves, with the GFP group serving as the control. (**B**) Detection and quantification of ROS signals using the luminol chemiluminescence assay, with the GFP group as the control.

To explore the function of the TaProDH1 gene, the GFP and TaProDH1-GFP constructs were transformed into *N. benthamiana* leaves via Agrobacterium-mediated infiltration. The two constructs were injected into different regions of the same *N. benthamiana* leaf, with the region expressing GFP alone serving as the control. The leaves were incubated in darkness for 10–12 h, followed by cultivation under normal light for 2–3 days. The leaves were harvested, and partial leaf tissues were used to observe GFP and TaProDH1-GFP signals under a fluorescence microscope. Leaves stained with DAB and trypan blue were used to detect ROS and cell death (Figure 3A). ROS react with luminol to form excited-state products (Figure 3B). Chemiluminescence values measured by a microplate reader were used to indirectly reflect the content of reactive oxygen species. The results indicated that the expression of TaProDH1 induced a significant accumulation of reactive oxygen species (ROS) compared with the control.

### 3.5. TaProDH Reduces Proline Content in N. benthamiana

**Figure 4:**
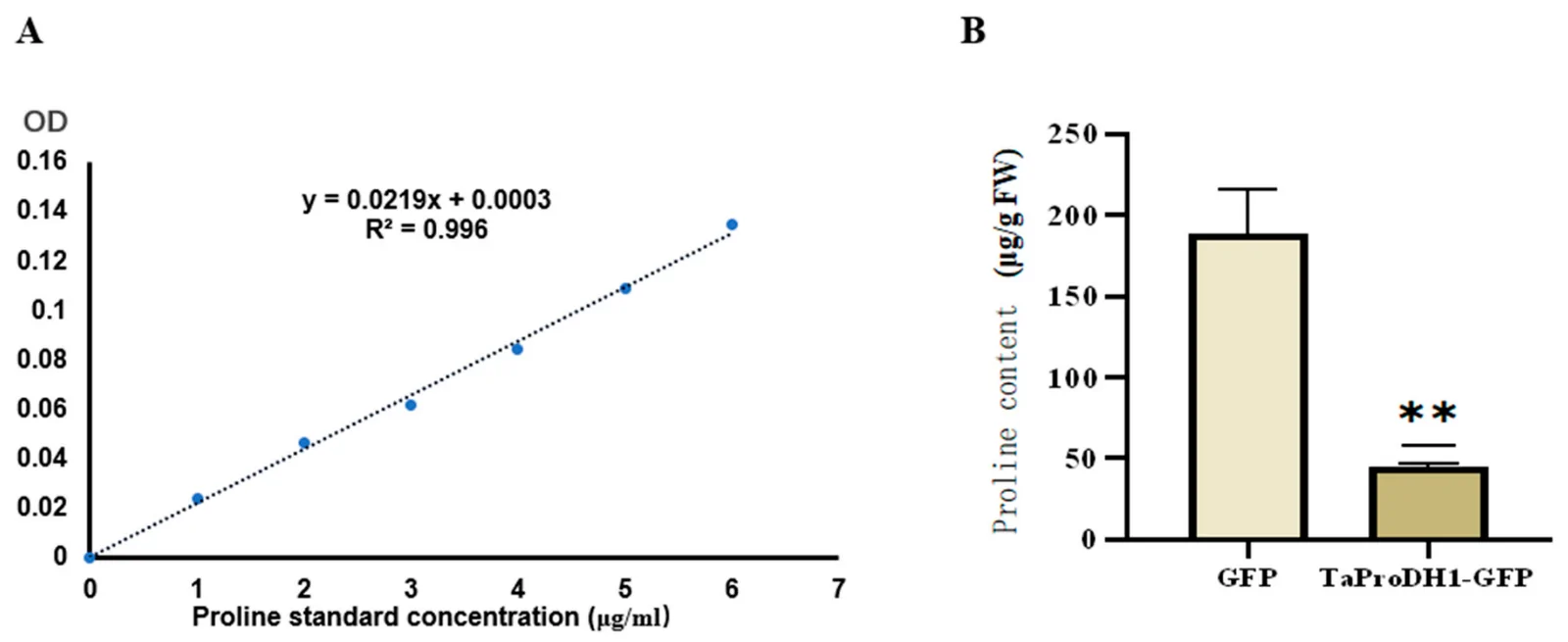
Determination of proline content after transient overexpression of TaProDH1 gene. (**A**) Establishment of proline standard curve. The abscissa represents the concentration of L-Pro standard solution (μg/mL), and the ordinate refers to the OD values of standards in a 96-well plate measured by a microplate reader at a wavelength of 600 nm. Regression equation: (y = 0.0219X + 0.0003), (R^2^ = 0.996). (**B**) Proline contents of GFP and TaProDH1-GFP. Unit μg/g FW indicates proline content per gram fresh weight of sample. Error bars represent three biological replicates, and double asterisks indicate (*p* < 0.01).

We also investigated whether TaProDH1 affects intracellular proline levels. The constructs pCAMBIA1302-*GFP* and *TaProDH1*-pCAMBIA1302 were transformed into Agrobacterium tumefaciens and subsequently infiltrated into *N. benthamiana*. A reliable standard curve was established, with a coefficient of determination (R^2^) of 0.996 (Figure 4A). Fresh leaf samples (1.0 g each) from plants expressing GFP and TaProDH1-GFP were collected for proline quantification. The results showed that the proline content in the TaProDH1-GFP group decreased by 75% compared with the control (Figure 4B).

### 3.6. Silencing of TaProDH1 in Wheat Reduces Its Resistance to the Stripe Rust Fungus

**Figure 5:**
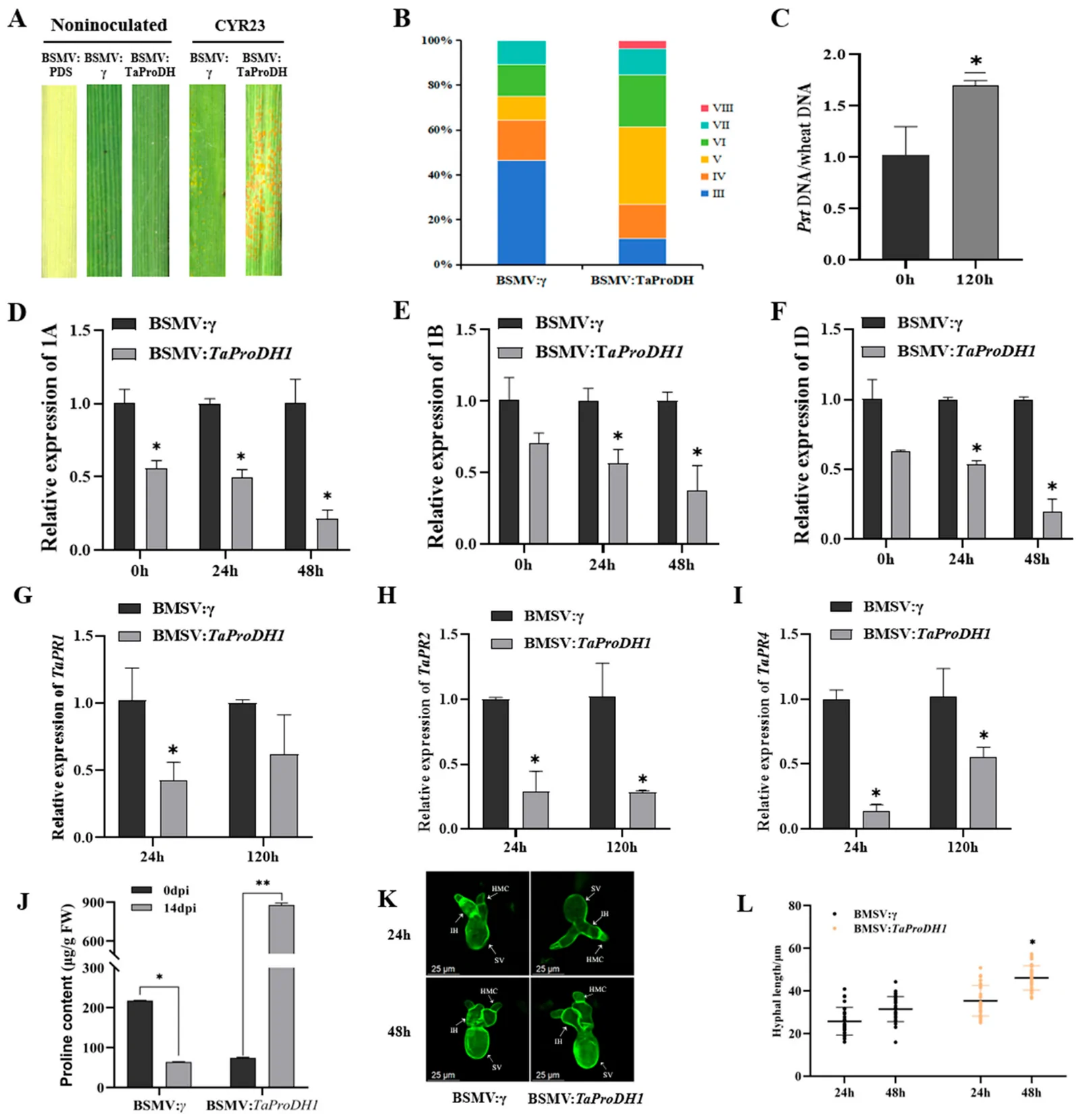
Functional analysis of VIGS transient silencing of the *TaProDH1* gene. (**A**) Phenotypes upon pathogen inoculation after silencing the *TaProDH1* gene. BSMV refers to barley stripe mosaic virus, and γ*-PDS* encodes wheat phytoene desaturase. (**B**) Stripe rust disease grading scale of wheat inoculated with *Puccinia striiformis* f. sp. *tritici* race CYR23 after silencing the *TaProDH1* gene. (**C**) Fungal biomass in wheat infected by stripe rust fungus at 14 dpi. (**D**–**F**) qRT-PCR analysis of TaProDH1 silencing efficiency at 24 h and 120 h after silencing treatment. (**G**–**I**) the expression patterns of TaPR1, TaPR2 and TaPR4 detected by qRT-PCR after TaProDH1 gene silencing, respectively. (**J**) Measurement of proline content in wheat infected with *Puccinia striiformis* f. sp. *tritici* at 14 days post inoculation (14 dpi). (**K**) Development of *Pst* hyphae stained with WGA (SV: substomatal vesicle; HMC: haustorial mother cell; IH: invasive hyphae). Scale bar = 25 μm. (**L**) Hyphal length at 24 h and 48 h after inoculation with CYR23. Error bars represent three biological replicates, and asterisks indicate significant differences between the treatment group and the control group (*, *p* < 0.05 and **, *p* < 0.01).

To explore whether *TaProDH1* is involved in regulating wheat resistance against *Pst*, we employed a BSMV-VIGS assay. Specific fragments were designed to specifically silence the *TaProDH1* genes (TaProDH1-1A/1B/1D) in wheat Su11. Wheat seedling leaves were inoculated separately with BMV:γ (negative control), BSMV:γ-*PDS* and BSMV:*TaProDH1* (silenced plants). Mild photobleaching was observed on leaves inoculated with BSMV:γ-*PDS* at 14 t(dpi), while no apparent growth defects were observed, proving the effectiveness of the VIGS system (Figure 5A).

The fourth leaves of gene-silenced wheat plants were inoculated with the incompatible race CYR23. Disease phenotypes were assessed at 14 dpi. Compared with the BSMV:γ control group, the BSMV:*TaProDH1*-silenced plants exhibited reduced resistance to CYR23 (Figure 5B). In addition, the biomass of stripe rust fungus in leaves of *TaProDH1*-silenced plants was significantly higher than that in the control leaves (Figure 5C). The relative expression of TaProDH1 was detected by qRT-PCR after *TaProDH1* gene silencing, before *Pst* inoculation and during *Pst* infection—labeled as 0, 24, and 48 hpi (Figure 5D–F). We analyzed the expression levels of defense-related genes *TaPR1*, *TaPR2* and *TaPR4* in BSMV:*TaProDH1*-silenced plants and BSMV:γ control plants. At 24 h after inoculation with stripe rust race CYR23, the transcript levels of these three genes were all markedly lower than those of the control (Figure 5G–I).

We measured proline contents in BSMV: *TaProDH1*-silenced plants and BSMV:*γ* control plants at 14 days after *Pst* infection. The proline level in the leaves of silenced plants was significantly higher than that in the control plants (Figure 5J). The proline content of control plants decreased after inoculation, while a notable increase was detected in silenced plants. Histological observation was performed to monitor the growth and development of *Pst* hyphae. At 48 hpi, the hyphal length of the pathogen in *TaProDH1*-silenced leaves was significantly longer than that in the control (Figure 5K,L). Silencing of *TaProDH1* promoted the growth and development of *Pst* in wheat leaves. In conclusion, silencing of *TaProDH1* reduced wheat resistance to stripe rust by downregulating the expression of *PR* genes and promoting the development of hyphae. *TaProDH1* positively regulates wheat resistance to *Pst*.

### 3.7. Screening for Upstream Transcription Factors Regulating the Expression of TaProDH1

**Figure 6:**
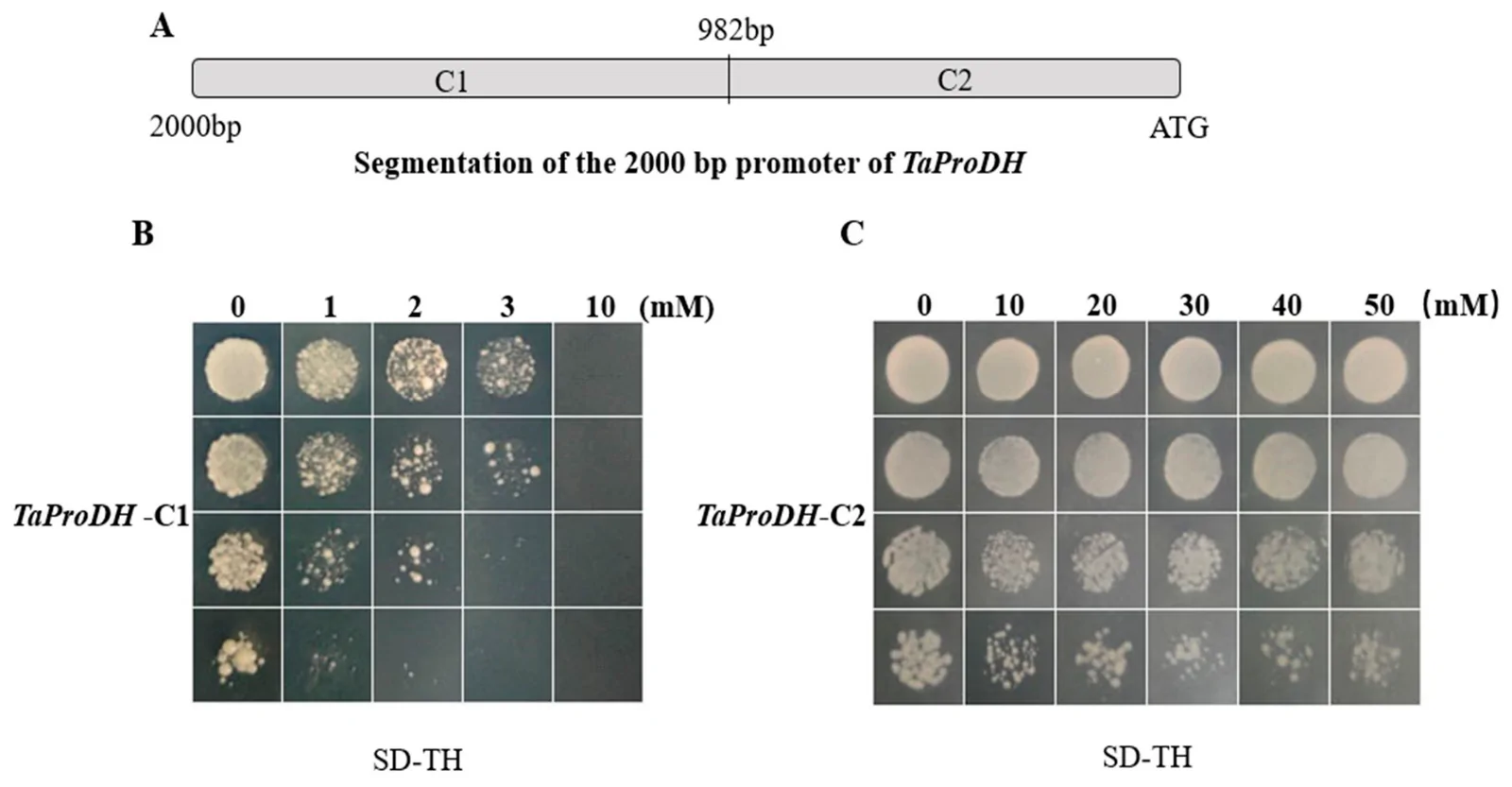
Screening of 3-AT concentrations for inhibiting self-activation of the TaProDH1 promoter. (**A**) Schematic diagram of truncated fragments of pHis2-*TaProDH1*:2000pro. (**B**) Yeast suspensions harboring the pHis2-*TaProDH1-C1* vector were adjusted to OD values of 0.2, 0.02, 0.002 and 0.0002, then spotted onto SD-Trp/His dropout plates supplemented with 0, 1, 2, 3 and 10 mM 3-AT. Yeast growth was observed after incubation at 28 °C for 3–4 days. (**C**) Spotting assay of pHis2-TaProDH1-C2 on SD-Trp/His dropout plates containing 0, 10, 20, 30, 40 and 50 mM 3-AT, with cultivation at 28 °C for 3–4 days to record yeast growth status.

To further elucidate the molecular regulatory network of TaProDH1 in wheat resistance to stripe rust, we performed Y_1_H assays to screen upstream transcription factors that interact with TaProDH1. We performed an autoactivation assay on the 2000 bp promoter of *TaProDH1,* screening for the optimal concentration of 3-AT to suppress the autoactivation of the 2000 bp *TaProDH1* promoter. Yeast harboring the 2000 bp *TaProDH1* promoter still grew when supplemented with 100 mM 3-AT, indicating strong autoactivation activity (Figure S4). Subsequently, we truncated the promoter fragment at 982 bp upstream of the start codon. Autoactivation inhibition assays were performed on the truncated constructs pHis2-*TaProDH1*-*C1* pro and pHis2-*TaProDH1-C2* pro (Figure 6A). The optimal 3-AT concentration for suppressing the autoactivation of pHis2-*TaProDH1-C1* pro was 3 mM (Figure 6B). The *TaProDH1-C1* promoter was selected for target screening assays. The pHis2-*TaProDH1-C1* promoter was constructed as bait, and the yeast one-hybrid assay was used to screen transcription factors. Single yeast colonies grown on SD-Trp/His/Leu medium supplemented with 3.5 mM 3-AT were picked for sequencing, and the sequencing data were further analyzed. We performed BLAST analysis on the five screened candidate sequences using the BLAST tool on the Ensembl Plants website (https://plants.ensembl.org/Multi/Tools/Blast, accessed on 16 July 2026) (Table 1). As a result, the transcription factor MYB30 was identified.

### 3.8. Interaction Between the TaProDH1 Promoter and the Transcription Factor TaMYB30

To confirm that TaMYB30 is a transcription factor localized in the nucleus, we conducted subcellular localization of TaMYB30. The TaMYB30 gene fused with the eGFP vector and the eGFP vector alone (control) were separately transformed into *N. benthamiana* leaves and wheat protoplasts. After analyzing the fluorescence microscopy observations, green fluorescence was detected in both the nucleus and the cytoplasm expressing GFP, while fluorescence was exclusively localized to the nucleus in leaves expressing TaMYB30-GFP. Therefore, we conclude that TaMYB30 is a nuclear protein (Figure S3).

**Figure 7:**
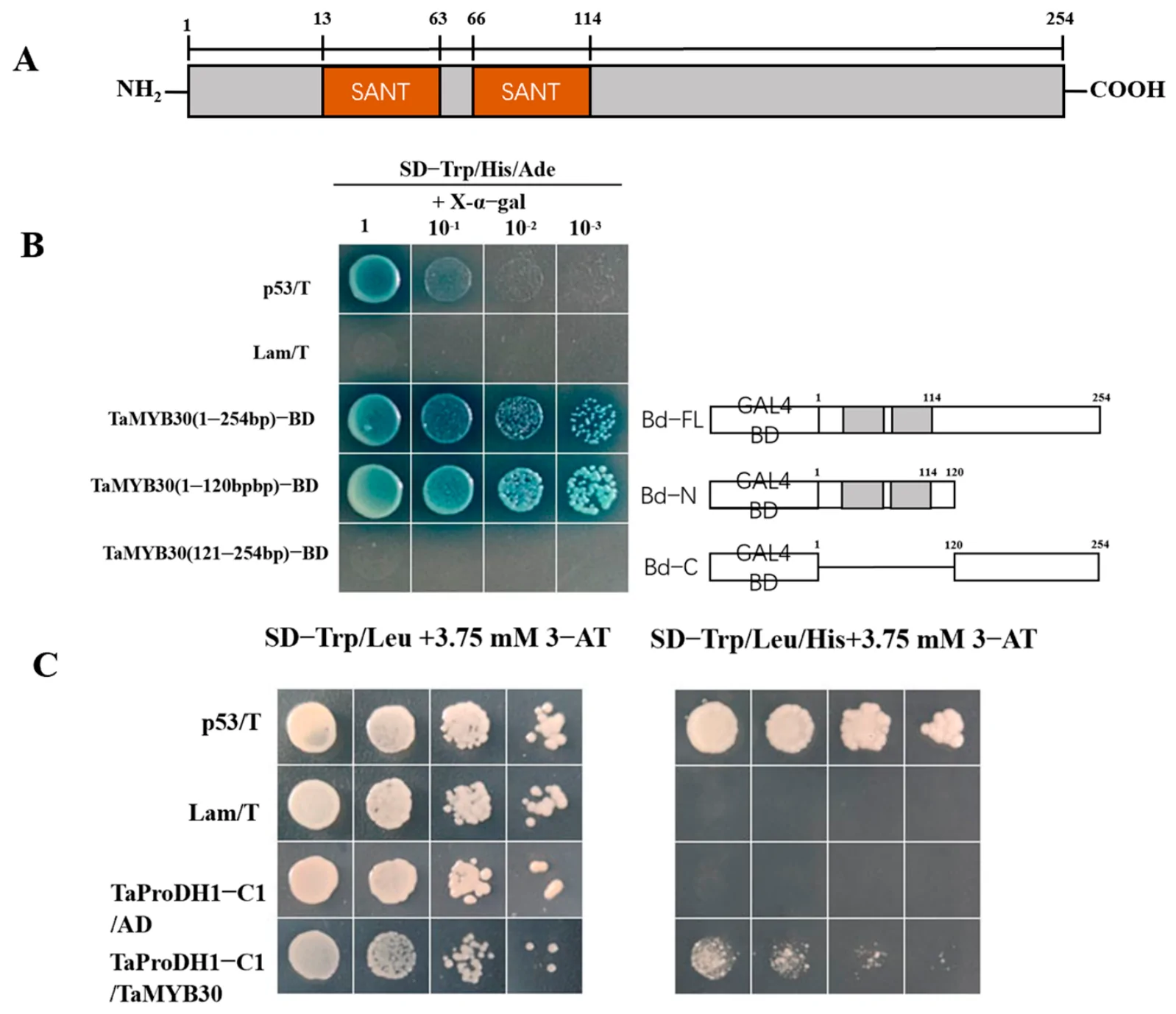
TaMYB30 encodes an MYB-family transcriptional activator. (**A**) Schematic diagram of the domain structure of TaMYB30. (**B**) Identification of the transcriptional activation region in TaMYB30 and schematic diagram of its truncated segments. (**C**) Interaction between transcription factor TaMYB30 and the TaProDH1-C1 (983–2000 bp) promoter. SD-Trp/Leu: dropout medium lacking tryptophan and leucine, used to verify the coexistence of TaMYB30-pGADT7 and TaProDH1-C1-pro. SD-Trp/Leu/His: dropout medium lacking tryptophan, leucine and histidine.

To identify whether the TaMYB30 protein is a transcriptional activator or repressor, we analyzed the protein sequence of TaMYB30 and identified two conserved SANT domains, which are located at amino acid residues 13–63 and 66–114, respectively (Figure 7A). To further verify that TaMYB30 is a transcriptional activator whose activation activity is mediated by its conserved SANT domains, the full-length TaMYB30 and its truncated constructs were cloned into the pGBKT7 vector and expressed in yeast cells. The full-length TaMYB30 and the fragment containing only amino acid residues 121–254 were able to grow on SD-Trp/His/Ade dropout medium, while the fragment covering amino acid residues 1–120 failed to grow on this SD-Trp/His/Ade-deficient medium (Figure 7B). These results demonstrate that TaMYB30 functions as a transcriptional activator, and it is inferred that the transcriptional activation domain required for its biological function is located in the C-terminal region of TaMYB30.

For the Y2H assay verifying the interaction between TaMYB30 and TaProDH1, the full-length CDS of TaMYB30 was cloned into the pGADT7 vector and co-transformed into yeast cells with the TaProDH1-C1 recombinant construct. The co-transformed yeast grew normally on SD-Trp/Leu/His dropout medium supplemented with 3.75 mM 3-AT, while yeast co-transformed with the empty pGADT7 vector and the TaProDH1-C1 construct failed to grow on the same selective medium (Figure 7C).

### 3.9. Transcription Factor TaMYB30 Activates the Promoter of TaProDH1

**Figure 8:**
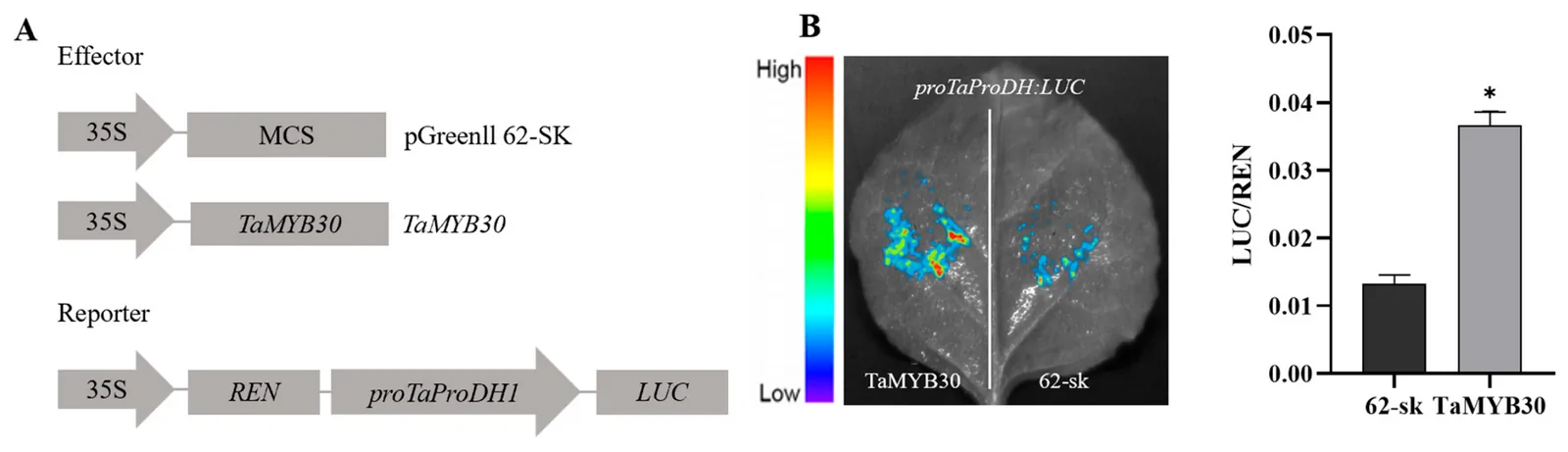
TaMYB30 activates the expression of *TaProDH1*. (**A**) Schematic diagram of effector and reporter constructs used for transient dual-luciferase assays. The full-length CDS of TaMYB30 was inserted into pGreenII 62-SK to generate the effector construct, while the 2 kb promoter of *TaProDH1* was cloned into pGreenII 0800-LUC to produce the reporter construct. (**B**) Dual-luciferase assay results indicate that TaMYB30 transcriptionally activates TaProDH1. LUC: firefly luciferase, serving as the experimental reporter gene; REN: Renilla luciferase, acting as the reference reporter gene. Asterisks indicate significant differences between the treatment group and the control group (*, *p* < 0.05).

To determine the regulatory function of TaMYB30 on TaProDH1, we performed a dual-luciferase assay on *N. benthamiana* leaves (Figure 8). Quantitative analysis of luciferase activity revealed that TaMYB30 could activate the expression of TaProDH1 (Figure 8B).

## 4. Discussion

Proline dehydrogenase (ProDH) serves as a pivotal rate-limiting enzyme in proline catabolism and has been widely implicated in plant growth, development, and adaptation to diverse environmental stresses. Currently, the molecular role of ProDH underlying the interaction between wheat and stripe rust fungus remains poorly characterized. We observed that TaProDH1 is targeted to the mitochondria, resembling the subcellular localization pattern of AtProDH1 in *Arabidopsis thaliana* [27]. Transient overexpression of *TaProDH1* induced a ROS burst in *N. benthamiana* leaves, which resembles H_2_O_2_ accumulation upon *OsProDH1* overexpression in rice [28], but is distinct from oxidative damage derived from proline overaccumulation in Arabidopsis *AtProDH1* mutants [29]. This difference may arise from species-specific ROS regulatory networks. Silencing *TaProDH1* markedly repressed ROS accumulation yet resulted in a complete loss of resistance. Transient overexpression elevated ROS content but failed to further strengthen disease resistance. These findings suggest that ROS thresholds are subject to stringent regulation; excessive ROS may trigger cell death and offset the beneficial effects on disease resistance. Further construction of stable transgenic silenced materials is required to elucidate the regulatory relationship among *TaProDH1*, ROS, and proline.

Analysis of the expression profiles of *TaProDH1* during stripe rust infection and its loss-of-function phenotypes demonstrated that *TaProDH1* positively regulates wheat immune resistance against stripe rust. RT-qPCR analysis showed that *TaProDH1* expression was significantly induced in wheat following inoculation with *Puccinia striiformis* f. sp. *tritici* races CYR23 and CYR34, with expression peaks occurring at 48–120 h post inoculation. This dynamic induction pattern is consistent with the PAMP-triggered expression signature of *AtProDH1* in Arabidopsis [10], implying that plants employ a conserved metabolic reprogramming strategy to coordinate immune responses. Accumulating evidence has indicated that proline metabolism plays dual regulatory roles in plant immunity. As an osmoprotectant and free radical scavenger, proline alleviates pathogen-induced oxidative damage [30]. Meanwhile, proline-derived degradation products actively participate in immune signal transduction [31]. The pathogen-triggered induction of *TaProDH1* was tightly synchronized with infection progression, whereas genetic loss of *TaProDH1* function completely compromised wheat disease resistance. Collectively, these results indicate that *TaProDH1* primarily mediates disease resistance via metabolism-to-signal transition.

The BSMV-VIGS system has been widely applied for the functional validation of wheat genes [24,32,33,34]. Virus-induced silencing of *TaProDH1* significantly compromised wheat resistance to the stripe rust race CYR23, as evidenced by accelerated lesion expansion and increased pathogen biomass. Combining our results with previous evidence, we propose a putative regulatory model whereby *TaProDH1* catalyzes proline degradation to generate P5C. Mitochondrial oxidation of P5C produces abundant NADH, which causes overload of the mitochondrial electron transport chain (ETC) and subsequent accumulation of O_2_^−^. The transient ROS burst further activates the MAPK cascade (e.g., *TaMPK3/6*) and induces the expression of defense-associated genes (e.g., *PR1* and *PAL*) [35]. This model is strongly supported by the enhanced ROS accumulation observed in *N. benthamiana* leaves transiently overexpressing *TaProDH1*. Nevertheless, the causal link between *TaProDH1*-mediated ROS dynamics and wheat disease resistance remains to be further validated in wheat. Notably, genetic knockdown of *TaProDH1* failed to completely eliminate host resistance, indicating that *TaProDH1*-dependent immunity may function synergistically with other defense signaling pathways, such as the SA and JA signaling cascades.

The MYB-family transcription factor TaMYB30 directly regulates the wheat proline dehydrogenase gene *TaProDH1*. Y1H assay results verified that TaMYB30 physically binds to the 983–2000 bp region of the TaProDH1 promoter, and dual-luciferase reporter assays further confirmed that TaMYB30 significantly enhances *TaProDH1* promoter activity. This regulatory pattern is consistent with the stress response mechanism mediated by *AtMYB44* in Arabidopsis, which functions by targeting the G-box cis-element (CACGTG) of downstream gene promoters [36]. TaMYB30 localizes to the nucleus and exhibits autonomous transcriptional activation, which is consistent with the canonical characteristics of transcriptional activators. Notably, unlike tomato SlMYB30, whose activation function exclusively depends on the C-terminal activation domain [37], the C-terminus of TaMYB30 is enriched in acidic amino acid residues (aspartic acid and glutamic acid). This structural feature may facilitate transcriptional activation by recruiting general transcription factors (e.g., TAF1) or histone acetyltransferases (HATs) [38]. Nevertheless, further biochemical validation, such as EMSA assays, is still required to confirm the direct binding of TaMYB30 to the *TaProDH1* promoter.

TaMYB30 positively regulates *TaProDH1* expression, thereby coupling transcriptional regulation with proline metabolic reprogramming and establishing a “transcription–metabolism–immunity” cascade in wheat. This regulatory module contributes to plant disease resistance through two core biological strategies. TaMYB30-mediated metabolic reprogramming provides essential energy and carbon resources for immune activation. TaMYB30 is rapidly induced during early pathogen infection and upregulates *TaProDH1* to accelerate proline catabolism. The released α-ketoglutarate enters the TCA cycle to boost ATP production and replenish carbon skeletons, which support multiple defense processes, including callose deposition and the secretion of pathogenesis-related proteins. This energy allocation strategy is analogous to the GmMYB29-dependent modulation of isoflavone biosynthesis in soybean plants, which enhances resistance against *Phytophthora* pathogens via metabolic reprogramming [39]. TaMYB30 coordinates the spatiotemporal dynamics of ROS signaling during immune responses. *TaProDH1*-driven proline degradation triggers robust ROS bursts, and TaMYB30 may further amplify this process via a positive feedback loop to sustain targeted ROS accumulation at infection sites [40], thereby ensuring effective local immune activation. Notably, this regulatory pathway is not independent but may be fine-tuned by additional upstream regulators. In Arabidopsis, the bZIP transcription factor HY5 competes with MYB30 for target promoter binding to balance light signaling and immune homeostasis [41]. Whether a similar antagonistic regulatory mechanism operates in wheat remains to be explored. Furthermore, TaMYB30 may maintain proline metabolic homeostasis by modulating other key metabolic genes, such as the proline synthesis-related gene *P5CS*, thereby dynamically balancing proline anabolism and catabolism during pathogen infection.

## Figures and Tables

**Table 1:** Partial sequencing results of the yeast one-hybrid screening library.

Number	ID	Candidate Sequence Name
1	TraesCS6D02G211400	Transcription factor MYB30 [Triticum aestivum]
2	TraesCS4D02G086400	chlorophyll a-b binding protein CP26, chloroplastic-like [Triticum aestivum]
3	TraesCS1B02G104600	uncharacterized protein LOC123111218 isoform X1 [Triticum aestivum]
4	TraesCS4B02G298900	hypothetical protein CFC21_058943 [Triticum aestivum]
5	TraesCS3A02G202700	probable protein phosphatase 2C 5 isoform X2 [Triticum aestivum]

## Data Availability

All data generated or analyzed in this research process are included in this published article and its Supplementary Materials.

## Supplementary Materials

The following supporting information can be downloaded at https://www.mdpi.com/article/10.3390/jof12090655/s1, Figure S1. Amino acid sequence alignment results of three copies of TaProDH1; Figure S2. Prediction and analysis of TaProDH1 expression induced by stripe rust; Figure S3. Subcellular localization of TaMYB30 protein; Figure S4. Screening of 3-AT concentration for inhibiting self-activation of *TaProDH1* promoter; Figure S5. Prediction of cis-acting elements of *TaProDH1* promoter.
