# Supplementary material for: Proline Dehydrogenase Gene TaProDH1 Positively Regulates Wheat Resistance to Stripe Rust Disease
Source: J Fungi (Basel). 2026 Sep 1;12(9):655. doi: 10.3390/jof12090655 (PMC13608731; doi:10.3390/jof12090655)
Supplement: Supplementary file 1 [file jof-12-00655-s001.zip › jof-4471780-supplementary.pdf]

```
TraesCS1D02G2124  MAIASRITKRALSTFAAAAKLPEAAVAAAGAEAVPAPSSAQHQQQQQVLEFEDTERLFTGEPSTALVRTLAALQLMSAGP  80
TraesCS1B02G2233  MAIASRITKRALSTFAAAAKLPEAAVAAAGAEAVPVPSSAQQQQ.HQVLEFEDTGRLFTGEPSTALVRTLAALQLMSAGP  79
TraesCS1A02G2091  ................................................................................   0
Consensus

TraesCS1D02G2124  LVDVGLAALRSPAVAASPVVQAAARATAYKHFCAGETADEAAARVQRLWRGGMGGILDYGIEDAEDGAACDRNVAGFLAA  160
TraesCS1B02G2233  LVDVGLAALRSPAVAASPVVQAAARATAYKHFCAGETADEAAARVQRLWRGGMGGILDYGIEDAEDGAACDRNVAGFLAA  159
TraesCS1A02G2091  ................................MRPRPGGRRGRRGRRRALLRMGGILDYGIEDAEDGAACDRNVAGFLAA   48
Consensus                                                    r  r     mggildygiedaedgaacdrnvagflaa

TraesCS1D02G2124  VDVAAALPPGSASVCIKITALCPIALLEKTSDLLRWQHKNPSVHLPWKQHAFPILSDSSPLYLTPSEPAALTAEEEHELQ  240
TraesCS1B02G2233  VDVAAALPPGSASVCIKITALCPIALLEKTSDLLRWQHKNPSVHLPWKQHAFPILSDSSPLYLTPSEPAALTAEEERELQ  239
TraesCS1A02G2091  VDVAAKLPPGSASVCIKITALCPIALLEKTSDLLRWQHKNPSVHLPWKQHAFPILSDSSPLYLTPSEPAALTAEEERELQ  128
Consensus         vdvaa lppgsasvcikitalcpiallektsdllrwqhknpsvhlpwkqhafpilsdssplyltpsepaaltaeee elq

TraesCS1D02G2124  LAHDRLLAVGARCAEHDIPLLVDAEYASVQPSIDYFTFVGALACNGGGRPIVHGTVQAYLRDARDRLEAMVRAAEEERVC  320
TraesCS1B02G2233  LAHDRLLAVGARCAEHDIPLLVDAEYASVQPSIDYFTFVGALACNGGGRPIVHGTVQAYLRDARDRLEAMVRAAEEERVC  319
TraesCS1A02G2091  LAHDRLLAVGARCAEHGIPLLVDAEYASVQPSIDYFTFVGALACNGGGRPIVHGTVQAYLRDARDRLEAMVRAAEEERVC  208
Consensus         lahdrllavgarcaeh ipllvdaeyasvqpsidyftfvgalacngggrpivhgtvqaylrdardrleamvraaeeervc

TraesCS1D02G2124  LGVKIVRGAYLTREARLAESLGVPSPIHGSIQDTHDCYNGCAAFLLERVRRGSASVMLATHNVESGQLAAARAQELGIGK  400
TraesCS1B02G2233  LGVKIVRGAYLTREARLAESLGVPSPIHGSIQDTHDCYNGCAAFLLERVRRGSASVMLATHNVESGQLAAARAQELGIGK  399
TraesCS1A02G2091  LGVKIVRGAYLTREARLAESLGVPSPIHGSIQDTHDCYNGCAAFLLERVRRGSASVMLATHNVESGQLAAARAQELGIGK  288
Consensus         lgvkivrgayltrearlaeslgvpspihgsiqdthdcyngcaafllervrrgsasvmlathnvesgqlaaaraqelgigk

TraesCS1D02G2124  GDRNLQFAQLMGMADGLSLGLRNAGFQVSKYLPYGPVEHIIPYLIRRAEENRGLLSASAFDRQLLRKELVRRFKNAVMGR  480
TraesCS1B02G2233  GDRNLQFAQLMGMADGLSLGLRNAGFQVSKYLPYGPVEHIIPYLIRRAEENRGLLSASAFDRQLLRKELVRRFKNAVMGR  479
TraesCS1A02G2091  GDRNLQFAQLMGMADGLSLGLRNAGFQVSKYLPYGPVEHIIPYLIRRAEENRGLLSASAFDRQLLRKELVRRFKNAVMGR  368
Consensus         gdrnlqfaqlmgmadglslglrnagfqvskylpygpvehiipylirraeenrgllsasafdrqllrkelvrrfknavmgr
```

Figure S1. Amino acid sequence alignment results of three copies of TaProDH1

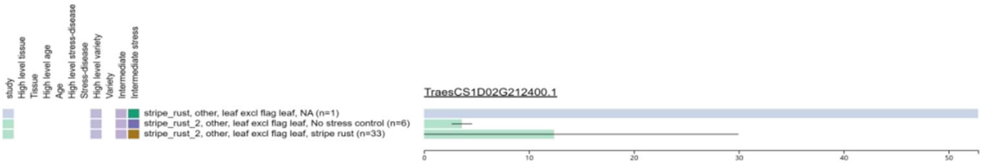

Figure S2. Prediction and analysis of TaProDH1 expression induced by stripe rust

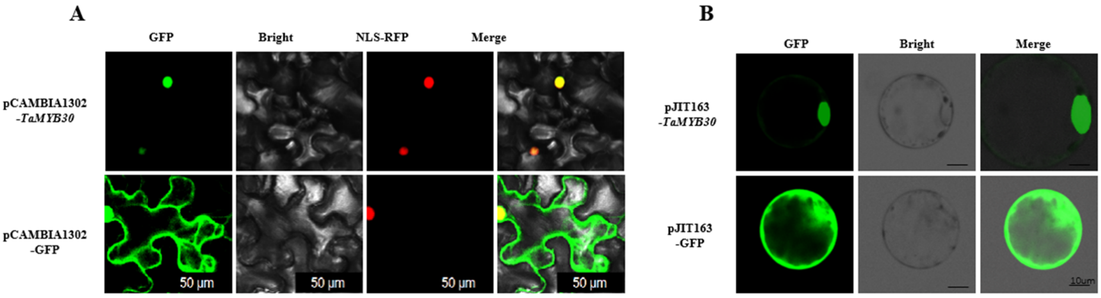

Figure S3. Subcellular localization of TaMYB30 protein.

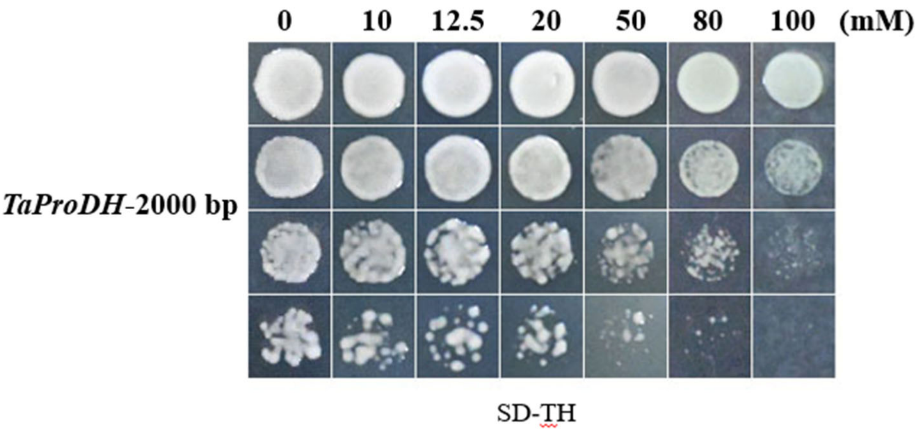

Figure S4 Screening of 3-AT concentration for inhibiting self-activation of TaProDH1 promoter

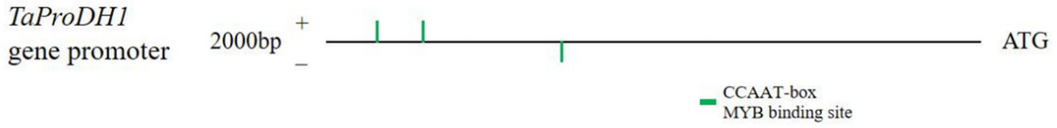

Figure S5 Prediction of cis-acting elements of TaProDH1 promoter
